# Implementation and use of electronic patient records in the Brazilian Air Force: a cross-sectional study^1^

**DOI:** 10.1590/1806-9282.20231136

**Published:** 2024-05-03

**Authors:** Patrícia Mesquita Vilas Boas, Luiza Jane Eyre de Souza Vieira, Geraldo Bezerra da Silva, Geison Vasconcelos Lira, Juliana Gomes Ramalho de Oliveira

**Affiliations:** 1 Universidade de Fortaleza – Fortaleza (CE), Brazil.; 2 Universidade Federal do Ceará – Sobral (CE), Brazil.

**Keywords:** Electronic health records, Health information systems, Health planning

## Abstract

**OBJECTIVE:**

The objective was to analyze the implementation and use of the electronic patient record in the health services of the Brazilian Air Force.

**METHODS:**

This is a cross-sectional study carried out with 234 physicians, between March and May 2021. The data collection instrument was sent by email. The electronic patient record was implemented in the Air Force approximately 3 years ago (64.5%), and about 81% of the physicians received training to operate it.

**RESULTS:**

The most common records involve data related to consultations (90.1%) and interviews with physical examination (67.1%). Physicians cited that information storage (75.6%), agility, and feasibility of recording (55.1%) were the main advantages of the electronic patient record. As disadvantages, problems in electronic equipment (69.7%) and system errors (65%) were reported. Most participants considered that the implementation had a positive impact on work dynamics (75.6%) and productivity (66.7%), mainly regarding the components "Work processes" (57.3%) and "Amount of carried out activities" (21.4%). Keeping records was significantly associated with the job position (p<0.001), type of unit (p=0.008), time of implementation (p<0.001), and participation in training (p=0.028).

**CONCLUSION:**

The implementation of the electronic patient record in the Air Force was recently done, and just over half of the physicians were trained prior to the implementation. The tool is considered compatible with work processes and has a positive effect on productivity.

## INTRODUCTION

The electronic patient record (EPR) is defined by the Federal Council of Medicine as a document consisting of information generated from facts, events, and situations related to the patient's health and the care provided, which has a legal, confidential, and scientific characteristic, in addition to allowing communication between members of the multidisciplinary team^1^.

The implementation of the EPR in Brazilian territory is regulated by Laws 13,709 and 13,787 of 2018; however, it is not widely disseminated in public and private health systems. These laws regulate personal data protection and patient records handling. The former is the base for all the sectors of our society regarding personal data, including health data and data from patients. The latter is the base for digitization and use of computerized systems for the custody, storage, and handling of patient records and are available at https://www.planalto.gov.br/ccivil_03/_ato2015-2018/2018/lei/l13709.htm and https://www.gov.br/conarq/pt-br/legislacao-arquivistica/leis-e-decretos-leis/lei-no13-787-de-27-de-dezembro-de-2018#:∼:text=Disp%C3%B5e%20sobre%20a%20digitaliza%C3%A7%C3%A3o%20e,manuseio%20de%20prontu%C3%A1rio%20de%20paciente. From the creation of the e-SUS Primary Care system, which seeks to computerize Primary Health Care, and the publication of the User Manual of the Citizen Electronic Record System, Brazil has strengthened implementation actions in all regions^2^. It is noteworthy that the implementation and the adequate use of the EPR increase the quality and organization of health care, integrating the professionals involved in patient care and subsidizing a shared and more assertive health management^3^.

The Armed Forces are permanent and regular national institutions, organized hierarchically, consisting of the Navy, the Army, and the Air Force, which are intended to defend the Homeland and guarantee constitutional powers. The Air Force Health System (SISAU, Sistema de Saúde da Aeronáutica), implemented in 1976 and reformulated in 1983, carries out activities related to the diagnosis and prevention of diseases, conservation or health recovery, and rehabilitation, covering all services provided in hospitals, offices, specialized clinics, and laboratories, or in home care.

Considering the advantages related to the use of EPR in health institutions and the importance of continuous improvement processes in care, the aim of this study was to analyze the implementation and use of EPR in the health services of the Brazilian Air Force. The main hypothesis of this study is that EPR, as a beneficial tool in the process of care, improves physicians’ work as well as improves information registration and increases security for patients.

## METHODS

### Study design and location

This is a cross-sectional study carried out at the Air Force Health System (SISAU).

### Study population and sample

The population consisted of individuals who are currently commissioned Air Force physicians.

The Brazilian Air Force currently has 1,767 doctors as its staff. Of these, 598 work in health care services where EPR has already been implemented. This information was considered to calculate the sample size of the study, according to the formula for cross-sectional studies with cross-sectional outcomes and finite population, as presented below:


$$n=\frac{Z_{a/2}^{2}P\cdot (1-P)\cdot N}{Z_{a/2}^{2}P\cdot (1-P)+(N-1)\cdot e^{2}}$$

Confidence level=99%

n=598

p=0.5

e=5%

The final sample size, defined by sample calculation, included 234 participants.

### Data collection

The study invitation was sent by email, between March and May 2021. The EPR was implemented in 2019, but there were no records of evaluations of the implementation stages and the impact of PEP on the work process.

The data collection instrument was developed using the Google Forms^®^ platform, based on the critical components of the EPR implementation pointed out in the literature^4^, and consisted of four blocks: sociodemographic characteristics, training and professional experience, conditions for EPR operability and impacts on work dynamics and potentialities, and challenges in the implementation.

The Google Forms^®^ platform has general data protection regulation (GDPR) in accordance with resolution 466/2012, which regulates research with human beings in Brazil (https://conselho.saude.gov.br/resolucoes/2012/Reso466.pdf). In this policy, Google commits to encrypting data collected by the Forms^®^ feature at multiple levels, by forcing the adoption of hypertext transfer protocol secure (HTTPS) in all transmissions, and the use of perfect forward secrecy (PFS) in all services. It is also committed to encrypting message transmissions with other servers using Transport Layer Security (TLS) and encryption keys relevant to the validation and key exchange phases. These procedures protect messaging communications when respondents fill out and submit their information. With this, the company Google assures users of its Forms^®^ resource regarding the security of the data entered (https://policies.google.com/privacy?hl=en-US). The platform also has terms of use and privacy policies that guarantee the maintenance of data anonymity and non-sharing of collected information. The platform's GDPR, terms of use, and privacy policies can be accessed at https://www.termsfeed.com/blog/terms-conditions-google-forms/.

The first screen of the instrument contained the free and informed consent form (TCLE). In case of agreement, the subsequent screen was displayed. Otherwise, the participants viewed the screen that ended the contract. There were four blocks of questions, divided into the following sections:

Block I: sex, age, marital status, children, state/municipality where you live and work, personal/family income (7 questions);

Block II: time since graduation, institution completing course, postgraduate degree, area of specialization, working time/weekly workload in aeronautics, time using EPR, use of other forms of records, abandonment of registration in EPR, work in another location… (17 questions);

Block III: EPR implementation time, training, duration, content, confidence in the EPR, time spent on records, satisfaction… (21 questions);

Block IV: potentials and challenges in implementing the patient's electronic medical record: speed, security, reliability, professionals responsible for adjustments to the EPR, interest, strengths and weaknesses (7 questions).

Data collection took place after approval by the Research Ethics Committee of the University of Fortaleza, under number 4,674,238.

### Data analysis

The data were exported to a Microsoft Excel^®^ spreadsheet (Microsoft, Albuquerque, New Mexico, USA, 1985–2003) and subsequently transferred to the Stata v.15 software (StataCorp LLC, College Station, Texas, USA, 1985–2017). Descriptive analysis was performed by calculating the position and dispersion measures for quantitative variables, and the frequencies for categorical variables. The inferential analysis began by checking the normality of quantitative variables using Shapiro-Wilk test. Mean difference tests were used to verify the existence of statistically significant differences between the means of numerical responses, depending on the distribution of categorical variables belonging to the blocks of the data collection instrument. For dichotomous variables, Student's t-test or Mann-Whitney test was applied. For polychotomous variables, a one-way ANOVA or Kruskal-Wallis test was applied, depending on the normality and variance of the data. For categorical variables, Pearson's chi-square test or Fisher's exact test was applied. The significance level was set at 5%.

## RESULTS

The study included 234 physicians, with a mean age of 39.25 (±7.53) years, predominantly female (56.8%), and who had graduated 14.17 (±7.58) years ago (Table 1). As for postgraduate studies, residency predominated (61.2%), work and reside in the same municipality (93.6%), average work experience of 9.82 (±7.82) years in hospitals (55. 5%) and in the Health Squad (34.1%), and an average workload of 33.70 (±10.86) h per week.

**Table 1:** Sociodemographic and occupational characteristics of the physicians in the Brazilian Air Force.

Variables		n	%
Sex			
	Female	133	56.8
	Male	101	43.2
Age range (years)			
	≤35	85	36.3
	≥35	149	63.7
Rank in the Air Force			
	Lieutenant	120	51.2
	Captain	60	25.6
	Major	36	15.4
	Colonel	10	4.3
	Lieutenant colonel	8	3.5
Time since graduation (years)			
	<5	31	13.2
	6–20	162	69.2
	>20	41	17.6
Work experience as a physician in the Air Force (years)			
	Up to 5 years	99	42.3
	6–15 years	86	36.8
	16 years or more	49	20.9
Type of unit where they work			
	Health Squad	85	36.3
	Hospital	130	55.6
	Other	19	8.1

Fortaleza, CE, Brazil, 2021 (n=234). Source: study data.

According to the participants, the EPR was implemented in the Air Force 3 years ago or less (64.5%). About 81% received training to operate it and the most present contents were access to the system (70.9%), filling out (70.0%), and medical record items (65.8%).

The EPR records were carried out by approximately 90% of the physicians. However, a significant number stopped using it at some point, mainly due to structural reasons (Table 2).

**Table 2:** Characterization of the implementation and use of the electronic patient record in the health services of the Brazilian Air Force.

Implementation				Use			
Variables		n	%	Variables		n	%
Time				Time			
	≤3 years	151	64.5		≤2 years	172	73.5
	>3 years	66	28.2		>2 years	62	26.5
	Does not know	10	4.3	EPR records			
	Under implementation	7	3.0		Yes	211	90.2
Participation in training					No	9	3.9
	Yes	190	81.2		Sometimes	14	5.9
	No	44	18.8	Did you stop using it at some point?			
Duration of training					Yes	171	73.1
	Up to 1 month	169	88.9		No	63	26.9
	Up to 6 months	6	3.2	Reasons for not using EPR			
	EPR records	3	1.6		Structural	151	88.4
	Does not know	12	6.3		Work dynamics	10	5.8
Training before the implementation					Other	10	5.8
	Yes	131	56.0				
	No	103	44.0				

Fortaleza, CE, Brazil, 2021 (n=234). Source: study data.

The purposes of using the EPR were consultations (90.1%), interviews and physical examinations (67%), evolution (62.8%), prescriptions (38%), discharge (28.2%), admission (18.8%), and requesting tests (1.3%). Information storage (75.6%), agility, and feasibility of recording (55.1%) were the main advantages of the EPR. As disadvantages, the following were reported: problems in electronic equipment (69.7%) and system errors (65%).

As for satisfaction with EPR use, 56.8% considered themselves satisfied with the implementation, 56.4% with quality, 51.7% with security, and 58.1% with usability. Most participants considered that the implementation had a positive impact on work dynamics (75.6%) and productivity (66.7%), mainly regarding the components "Work processes" (57.3%) and "Amount of carried out activities" (21.4%).

As for the advantages of using the EPR, agility and feasibility were mentioned by 55.1% of the participants and storage of information by 75.6%. However, for 66.7% there was no increase in the efficiency of care, 91% did not observe team satisfaction, and 90.6% of physicians did not relate the EPR to a better relationship with patients. The main strengths were the EPR as a care tool (99.4%), the health professionals’ interest (71.4%), and the quality of the EPR (70.1%). As weaknesses, the time spent (55.1%) and the level of training (46.2%) were mentioned.

Data recording was significantly associated with the participants’ rank (p<0.001), type of unit where they are stationed (p=0.008), time of implementation (p<0.001), and participation in training (p=0.028) (Table 3).

**Table 3:** Association between sociodemographic and labor variables and data recording in the electronic patient record in the Brazilian Air Force.

Variables		Data recording in EPR			p-value
		Yes n (%)	No n (%)	Sometimes n (%)	
Rank in Air Force					<0.001†
	Lieutenant	112 (93.4)	1 (0.8)	7 (5.8)	
	Captain	55 (91.7)	1 (1.7)	4 (6.6)	
	Major	27 (75.0)	7 (19.4)	2 (5.6)	
	Colonel	10 (100.0)	0	0	
	Lieutenant colonel	7 (87.5)	0	1 (12.5)	
Type of unit where they work					0.008†
	Health Squad	73 (85.9)	3 (3.5)	9 (10.6)	
	Hospital	124 (95.4)	4 (3.1)	2 (1.5)	
	Other	14 (73.7)	2 (10.5)	3 (15.8)	
Time of EPR implementation at the unit					<0.001†
	Up to 6 months	37 (90.2)	1 (2.5)	3 (7.3)	
	6 months to 1 year	30 (85.7)	1 (2.9)	4 (11.4)	
	1–2 years	71 (94.6)	2 (2.7)	2 (2.7)	
	3–5 years	58 (95.1)	1 (1.6)	2 (3.3)	
	5 years or longer	0	4 (80.0)	1 (20.0)	
	Under implementation	1 (14.3)	4 (57.1)	2 (28.6)	
	Does not know	10 (100.0)	0	0	
Participation in training to operate EPR					0.028‡
	Yes	176 (92.6)	5 (2.6)	9 (4.8)	
	No	35 (79.5)	4 (9.1)	5 (11.4)	
Prior training to EPR implementation					0.733‡
	Yes	118 (90.1)	6 (4.6)	7 (5.3)	
	No	93 (90.3)	3 (2.9)	7 (6.8)	

Fortaleza, CE, Brazil, 2021 (n=234).

‡ Chi-square test.

† Fisher's exact test. Source: study data.

## DISCUSSION

The implementation of the EPR in the Brazilian Air Force has been a recent event. It was found that 44% of physicians did not receive any training before the implementation, and 19% did not participate in any training at any time. It is known that the lack of training is a threat to the implementation of the EPR and can increase the chances of errors, recording delays, reduced satisfaction, and acceptability^4,5^. This was confirmed by the association between participation in training and electronic records in clinical practice (p=0.028). The participants considered the level of training a weakness related to EPR use, which can interfere with the incorporation of the tool in daily activities^3^. This study advances the understanding of the use of EPR by the physicians in the Brazilian Air Force, which is still less explored in the national scientific research. Given the positive perception about the aspects of EPR in the institution, there has been a successful implementation of electronic health records. It is noteworthy that this study represents, until November 2021, the first investigation to evaluate the implementation and the use of EPR in the Brazilian Armed Forces.

This study showed that consultations, interviews, physical examinations, and evolution were the main EPR purposes used by the physicians. The central role that the use of these characteristics plays in optimizing work processes, increasing the quality of records, fostering interprofessional discussion, and encouraging the achievement of best health practices is highlighted^6^. However, there was a need to offer training aimed at using it in admissions, discharge, and drug prescriptions.

Although approximately 55% of the physicians reported that the EPR implementation resulted in agility and feasibility in data recording, 66.7% did not observe an increase in the efficiency of care. Therefore, although the EPR contributed to the work dynamics, the increase in efficiency, which is one of the objectives of its implementation, is not being fully achieved. The use of artificial intelligence mechanisms is a strategy that can encourage this scenario, as it expands the possibilities of effective decision-making^7,8^. While physicians may perceive the EPR as a tool that requires more time to make records, patients express satisfaction with the records in information systems with interoperability.

The use of EPR was considered relevant to increase the productivity by most physicians. However, in the study by Kaneko et al., no significant differences or decreases in productivity were observed when compared with the use of traditional paper recording^9^. Hence, the perception of the impact of the EPR on work processes must be periodically reassessed to support continuous improvement strategies.

The participants’ rank in the Air Force and the time since the EPR was implemented in the work unit were associated with record performing (p<0.001 and p<0.001, respectively). It was verified that the frequency of recording in the EPR increased according to the time of implementation in the unit (p<0.001). In this process, the expansion of its use is relevant as it generates new technical, professional, and ethical challenges^10^ for the improvement of the EPR and, consequently, greater inclusion into the service routine.

The Brazilian Air Force has the potential to improve and expand the implementation of the EPR, since the health professionals’ interest and the system quality were considered strengths by more than 70% of the participants in this study. Both aspects are considered crucial for the usability and operability success of the EPR^11^.

The results allowed mapping the usability characteristics of the EPR and existing strengths, which should be promoted. Additionally, aspects for continuous improvement were identified, such as corrections of system errors and problems in electronic equipment, aiming to increase operational efficiency, user satisfaction, and the quality of care.

One limitation of this study is the gathering of information through one professional category. However, it is noteworthy that the study promotes substantial progress in understanding the use of EPR in the Air Force, whose health care is still less explored in scientific research. Given the positive perception, the institution can be considered a national reference regarding the implementation of the EPR. It is noteworthy that this is the first study, up to the moment of its conclusion, to assess the implementation and use of the EPR in the Brazilian Armed Forces. Given that software and operability updates are dynamic and constant, it is possible that perceptions have changed over time. Each professional uses specific EPR functionalities, and perceptions about EPR may differ between the members of the healthcare team.

It is suggested that, based on the knowledge provided in this research, longitudinal and prospective studies should be carried out to compare the efficiency, satisfaction, patient safety, and quality of care in Brazilian health services that adopt manual and electronic records. This comparison is important for both public and private spheres, including, but not limited to, the Brazilian Armed Forces. Expanding the use of artificial intelligence to improve clinical decision-making in healthcare and optimize the healthcare services offered the relationship between EPR and big data complex sets of data through various techniques, which include machine learning, enabling the manipulation and application of data stored in EPR.

## CONCLUSION

The implementation of the EPR in the Brazilian Air Force is recent, and just over half of the physicians were previously trained to use it. The most frequent recordings are those related to consultations, interviews, physical examinations, and evolution, with underutilization of the system for admissions, discharge, and drug prescriptions.

According to the physicians’ assessment, the main advantages of the EPR are data storage, agility, and feasibility of the recording, whereas the disadvantages are equipment problems and system errors. The EPR is considered compatible with work processes and has a positive effect on productivity.

Future studies should investigate the efficiency, satisfaction, patient safety, and quality of care in health services that adopt manual and electronic data recording. In the Brazilian Air Force, it is suggested that EPR user satisfaction surveys should be carried out periodically, with a view to improvement.

Multiple possibilities for expanding the use of PEP in the Brazilian Armed Forces exist, mainly in the application of approaches that allow processing, understanding, and learning from health information recording systems. This scenario will be feasible with techniques, computational methods that employ methods of organization, interpretation, and recognition of patterns in health information registered in the Brazilian Armed Forces, which will contribute to promoting practices developed in its health system on the effectiveness of treatments and clinical decisions aligned with the Health Surveillance model in the Brazilian Unified Health System.

## References

[1] Conselho Federal de Medicina (2002). Resolução nº 1.638/02, de 9 de agosto de 2002.

[2] Ministério da Saúde (BR) (2019). E-SUS Atenção Básica: manual de uso do sistema com prontuário eletrônico do cidadão PEC – Versão 3.2.

[3] Toledo PPDS, Santos EMD, Cardoso GCP, Abreu DMF, Oliveira AB (2021). Electronic health record: a systematic review of the implementation under the national humanization policy guidelines. Cien Saude Colet.

[4] Aguirre RR, Suarez O, Fuentes M, Sanchez-Gonzalez MA (2019). Electronic health record implementation: a review of resources and tools. Cureus.

[5] Ting J, Garnett A, Donelle L (2021). Nursing education and training on electronic health record systems: an integrative review. Nurse Educ Pract.

[6] Hedian HF, Greene JA, Niessen TM (2018). The electronic health record and the clinical examination. Med Clin North Am.

[7] Chi EA, Chi G, Tsui CT, Jiang Y, Jarr K, Kulkarni CV (2021). Development and validation of an artificial intelligence system to optimize clinician review of patient records. JAMA Netw Open.

[8] Yang X, Mu D, Peng H, Li H, Wang Y, Wang P (2022). Research and application of artificial intelligence based on electronic health records of patients with cancer: systematic review. JMIR Med Inform.

[9] Kaneko K, Onozuka D, Shibuta H, Hagihara A (2018). Impact of electronic medical records (EMRs) on hospital productivity in Japan. Int J Med Inform.

[10] Evans RS (2016). Electronic health records: then, now, and in the future. Yearb Med Inform.

[11] Wolfe L, Chisolm MS, Bohsali F (2018). Clinically excellent use of the electronic health record: review. JMIR Hum Factors.

